# Inhibin Alpha Expression in Human Tumors: A Tissue Microarray Study on 12,212 Tumors

**DOI:** 10.3390/biomedicines10102507

**Published:** 2022-10-07

**Authors:** Sören Weidemann, Nessar Ahmad Noori, Maximilian Lennartz, Viktor Reiswich, David Dum, Anne Menz, Viktoria Chirico, Claudia Hube-Magg, Christoph Fraune, Ahmed Abdulwahab Bawahab, Christian Bernreuther, Ronald Simon, Till S. Clauditz, Guido Sauter, Andrea Hinsch, Simon Kind, Frank Jacobsen, Stefan Steurer, Sarah Minner, Eike Burandt, Andreas H. Marx, Till Krech, Patrick Lebok, Franziska Büscheck, Doris Höflmayer

**Affiliations:** 1 Institute of Pathology, University Medical Center Hamburg-Eppendorf, 20246 Hamburg, Germany; 2 Department of Pathology, Faculty of Medicine, University of Jeddah, Jeddah 23890, Saudi Arabia; 3 Department of Pathology, Academic Hospital Fuerth, 90766 Fuerth, Germany; 4 Institute of Pathology, Clinical Center Osnabrueck, 49074 Osnabrueck, Germany

**Keywords:** inhibin A (INHA), tissue micro array, immunohistochemistry, human tumors, cancer aggressiveness

## Abstract

As a result of its expression in corresponding normal cell types, inhibin alpha (INHA) is used as an immunohistochemical marker for adrenocortical neoplasms and testicular or ovarian sex cord stromal tumors. However, other tumors can also express INHA. To comprehensively determine INHA expression in cancer, a tissue microarray containing 15,012 samples from 134 different tumor types and subtypes was analyzed by immunohistochemistry. INHA positivity was found in 72 of 134 tumor categories, including 26 categories with ≥1 strongly positive case. A moderate to strong INHA positivity was found in 100% of 37 granulosa cell tumors of the ovary, 100% of 43 other sex cord stromal tumors of the ovary/testis, 100% of 31 granular cell tumors, 78.5% of 28 adenomas, 44% of 25 carcinomas of the adrenal cortex, and 46.7% of 15 pancreatic acinar cell carcinomas. At least a weak INHA positivity was seen in <33% of cases of 46 additional tumor entities. In summary, these data support the use of INHA antibodies for detecting sex cord stromal tumors, granular cell tumors, and adrenocortical neoplasms. Since INHA can also be found in other tumor entities, INHA immunohistochemistry should only be considered as a part of any panel for the distinction of tumor entities.

## 1. Introduction

The inhibin alpha subunit protein (INHA) is a member of the TGF-beta (transforming growth factor-beta) superfamily encoded by a gene located at 2q35 [1,2,3]. It combines with the A and B type proteins of the inhibin beta subunits to form inhibin protein complexes that negatively regulate the secretion of follicle-stimulating hormone (FSH) from the pituitary gland [4,5,6]. Inhibin has also been suggested to inhibit gonadal stromal cell proliferation and to possess a tumor suppressive activity [4].

Among normal tissues, INHA staining is found in adreno-cortical cells, Sertoli and Leydig cells of the testis, and the placenta [7]. Accordingly, inhibin alpha is currently used as an immunohistochemical marker for adrenocortical tumors and sex cord stromal tumors of the testis and the ovary [7,8,9]. However, a systematic analysis of inhibin alpha across human cancer types would be highly desirable to understand the diagnostic value of inhibin alpha detection. This is all the more pressing since other tumor entities have also been reported to express inhibin alpha across many tumor types, although the reported positivity rates are highly variable. For example, inhibin alpha positivity has been described in 41–100% of granulosa cell tumors of the ovary [10,11,12], 25–100% of adrenocortical carcinomas [13,14,15,16], 0–100% of mucinous carcinomas of the ovary [17,18,19,20], 0–63% of serous high-grade carcinomas of the ovary [12,21,22], 0–60% of Brenner tumors of the ovary [12,22,23], 0–75% of endometroid carcinomas of the ovary [12,18], and 0–16% of pheochromocytomas [14,15,16,24,25,26,27,28,29]. These conflicting data may be caused by the different antibodies, immunostaining protocols, and criteria used to determine INHA positivity in these studies.

To better understand the prevalence and significance of INHA expression in cancer, a comprehensive study analyzing a large number of neoplastic and non-neoplastic tissues under highly standardized conditions is needed. We therefore analyzed INHA expression in more than 15,000 tumor tissue samples from 134 different tumor types and subtypes, as well as 76 non-neoplastic tissue categories by immunohistochemistry (IHC) in a tissue microarray (TMA) format in this study.

## 2. Materials and Methods

### 2.1. Tissue Microarrays (TMAs)

Our normal tissue TMA was composed of 8 samples from 8 different donors for each of 76 different normal tissue types (608 samples on one slide). The cancer TMAs contained a total of 15,012 primary tumors from 134 tumor types and subtypes. The arrayed cancer samples were mainly of Caucasian origin and underwent surgery at the University Medical Center Hamburg-Eppendorf between 1992 and 2016. Detailed histopathological data were available for 2,351 colorectal adenocarcinomas, 192 neuroendocrine tumors, and 801 clear cell renal cell carcinomas. These tumors were distributed across 48 TMA blocks containing between 80 and 522 tissue spots with a diameter of 0.6 mm each. For a subset of 531 kidney cancer patients, clinical follow-up data were also accessible with a median follow-up time of 40 months (range 1−250). The composition of normal and cancer TMAs is described in the Results section. All samples were from the archives of the Institutes of Pathology, University Hospital of Hamburg, Germany, the Institute of Pathology, Clinical Center Osnabrueck, Germany, and the Department of Pathology, Academic Hospital Fuerth, Germany. Tissues were fixed in 4% buffered formalin and then embedded in paraffin. The TMA manufacturing process has been described earlier in detail [30,31,32]. In brief, one tissue spot (diameter: 0.6 mm) was transmitted from a tumor containing donor block in an empty recipient paraffin block. The use of archived remnants of diagnostic tissues for TMA manufacturing, their analysis for research purposes, and patient data were conducted according to local laws (HmbKHG, §12), and the analysis was approved by the local ethics committee (Ethics Commission Hamburg, WF-049/09). All work was carried out in compliance with the Helsinki Declaration.

### 2.2. Immunohistochemistry

Freshly cut TMA sections were immunostained on one day and in one experiment. Slides were deparaffinized and exposed to heat-induced antigen retrieval for 5 min in an autoclave at 121 °C in a pH 7.8 buffer. A primary antibody specific for inhibin alpha (recombinant rabbit, MSVA-561R, MS Validated Antibodies, GmbH, Hamburg, Germany) was applied at 37 °C for 60 min at a dilution of 1:100. Bound antibody was then visualized using the EnVision Kit (Agilent, Santa Clara, CA, USA; #K5007) according to the manufacturer’s directions. For the purpose of antibody validation, the normal tissue TMA was also analyzed using a ready-to-use anti-inhibin diagnostic antibody (monoclonal mouse anti-human inhibin α, clone R1, Agilent, Santa Clara, CA, USA, cat.# IR058) according to the protocol suggested by the manufacturer. In brief, following pH 9 antigen retrieval, the TMA slide was stained in a DAKO auto-stainer Link48 with a FLEX detection system. An experienced pathologist performed manual analysis of the stained TMA slides. Hematoxylin- and eosin-stained sections were used for comparison in cases of questionable tumor cell content. For tumor tissues, the percentage of positive neoplastic cells was estimated, and the staining intensity was semi-quantitatively recorded (0, 1+, 2+, 3+). For statistical analyses, the staining results were categorized into four groups. Tumors without any staining were considered negative. Tumors with 1+ staining intensity in ≤70% of tumor cells or 2+ intensity in ≤30% of tumor cells were considered weakly positive. Tumors with 1+ staining intensity in >70% of tumor cells, 2+ intensity in 31–70%, or 3+ intensity in ≤30% were considered moderately positive. Tumors with 2+ intensity in >70% or 3+ intensity in >30% of tumor cells were considered strongly positive.

### 2.3. Statistics

Statistical calculations were performed with JMP 14 software (SAS Institute Inc., Cary, NC, USA). Contingency tables and the chi²-test were performed to search for associations between INHA and tumor phenotype. Survival curves were calculated according to Kaplan–Meier. The log-rank test was applied to detect significant differences between groups.

## 3. Results

### 3.1. Technical Issues

A total of 12,212 (81%) of 15,012 tumor samples were interpretable in our tumor TMA analysis. Non-interpretable samples demonstrated a lack of unequivocal tumor cells or loss of the tissue spot during technical procedures. A sufficient number of samples (<3) of each normal tissue type was evaluable.

### 3.2. Inhibin Alpha in Normal Tissues

**Figure 1:**
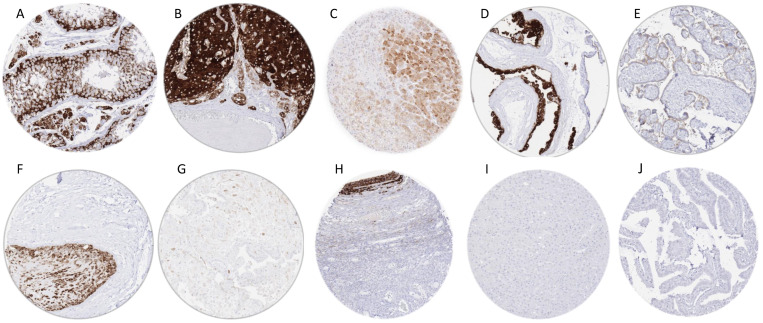
Inhibin alpha (INHA) immunostaining in normal tissues. The panels show an INHA immunostaining of variable intensity in Sertoli and Leydig cells of the testis (**A**), corpus luteum of the ovary (**B**), cortical cells of the adrenal gland (**C**), cytotrophoblast cells of the first trimester placenta (**D**), and of the mature placenta (**E**), chorion cells of the placenta (**F**), decidua cells in placenta adjacent tissue (**G**), and granulosa cells of follicular cysts of the ovary (**H**). INHA staining is completely absent in the liver (**I**) and the fallopian tube (**J**).

By using MSVA-561R, strong INHA staining was found in Sertoli and Leydig cells of the testis, corpus luteum of the ovary, and the cyto- and syncytiotrophoblast, as well as chorion cells of the placenta (stronger staining in the first trimester than in mature placenta), and in adrenocortical cells. A more variable staining intensity ranging from weak to strong was seen in the amnion and decidua cells of the placenta, as well as in follicular, granulosa, and some stroma cells of the ovary. Scattered INHA-positive epithelial cells were also seen in the pancreas and the adenohypophysis. Representative images of INHA-positive normal tissues are shown in Figure 1. All these cell types also stained positive if the monoclonal mouse anti-human inhibin alpha antibody clone R1 was used (Figure S1). INHA staining was not seen in any other analyzed tissues, including skeletal muscle, heart muscle, smooth muscle, myometrium, fat, transitional mucosa of the anal canal, urothelium of the renal pelvis and urinary bladder, lymph node, spleen, thymus, tonsil, mucosa of the stomach, duodenum, ileum, appendix, colon, rectum and gallbladder, liver, parotid gland, submandibular gland, sublingual gland, Brunner’s gland of the duodenum, kidney, prostate, seminal vesicle, epididymis, bronchial glands, lung, breast, endocervix, endometrium, fallopian tube, thyroid, parathyroid gland, cerebellum, cerebrum, and the neurohypophysis.

### 3.3. Inhibin Alpha in Cancer

Positive INHA immunostaining was detectable in 583 (4.8%) of the 12,212 analyzable tumors, including 351 (2.9%) with weak, 66 (0.5%) with moderate, and 167 (1.4%) with strong INHA positivity. Overall, 72 (54%) of 134 tumor categories showed detectable INHA expression, with 26 (19%) tumor categories including at least one case with strong positivity (Table 1).

The highest rate of positive staining and the highest levels of expression were found in various types of sex cord stromal tumors of the testis and the ovary (100% positive), granular cell tumors (100%), granulosa cell tumors of the ovary (100%), and adrenal cortical adenomas (93%) and carcinomas (80%), as well as in acinar cell carcinomas of the pancreas (80%). Sixty additional tumor entities showed INHA immunostaining not only less frequently but also at lower intensity. Of note, tumor cell nests in the ovary were often surrounded by a conspicuous layer of INHA positive stromal cells. This was independent of the tumor type. A comparison with the histopathologic parameters of cancer aggressiveness and/or clinical data revealed significant associations between INHA positivity and nodal metastasis in colorectal adenocarcinoma (*p* = 0.0494) and high Thoenes’ grade in clear cell renal cell carcinoma (*p* = 0.0498), as well as a tendency towards more nodal metastases in INHA-positive neuroendocrine tumors, although this relationship did not reach statistical significance (*p* = 0.0824; Table 2).

**Table 1:** Inhibin alpha (INHA) immunostaining in human tumors.

			Inhibin Alpha (INHA) IHC Result				
	Tumor Entity	On TMA (n)	Analyzable (n)	Negative (%)	Weak (%)	Moderate (%)	Strong (%)
**Tumors of the skin**	Pilomatrixoma	35	33	100.0	0.0	0.0	0.0
	Basal cell carcinoma	88	77	100.0	0.0	0.0	0.0
	Benign nevus	29	23	100.0	0.0	0.0	0.0
	Squamous cell carcinoma of the skin	90	83	100.0	0.0	0.0	0.0
	Malignant melanoma	46	41	100.0	0.0	0.0	0.0
	Malignant melanoma Lymph node metastasis	86	83	100.0	0.0	0.0	0.0
	Merkel cell carcinoma	46	39	100.0	0.0	0.0	0.0
**Tumors of the head and neck**	Squamous cell carcinoma of the larynx	109	101	95.0	5.0	0.0	0.0
	Squamous cell carcinoma of the pharynx	60	60	100.0	0.0	0.0	0.0
	Oral squamous cell carcinoma (floor of the mouth)	130	128	98.4	1.6	0.0	0.0
	Pleomorphic adenoma of the parotid gland	50	44	100.0	0.0	0.0	0.0
	Warthin tumor of the parotid gland	49	46	100.0	0.0	0.0	0.0
	Basal cell adenoma of the salivary gland	15	14	100.0	0.0	0.0	0.0
**Tumors of the lung, pleura, and thymus**	Adenocarcinoma of the lung	196	122	82.0	13.1	2.5	2.5
	Squamous cell carcinoma of the lung	80	48	95.8	4.2	0.0	0.0
	Small cell carcinoma of the lung	16	13	92.3	0.0	7.7	0.0
	Mesothelioma, epithelioid	39	23	91.3	4.3	4.3	0.0
	Mesothelioma, other types	76	53	98.1	1.9	0.0	0.0
	Thymoma	29	27	100.0	0.0	0.0	0.0
**Tumors of the female genital tract**	Squamous cell carcinoma of the vagina	78	53	100.0	0.0	0.0	0.0
	Squamous cell carcinoma of the vulva	130	123	99.2	0.8	0.0	0.0
	Squamous cell carcinoma of the cervix	128	124	98.4	1.6	0.0	0.0
	Adenocarcinoma of the cervix	21	21	100.0	0.0	0.0	0.0
	Endometrioid endometrial carcinoma	236	217	88.5	10.1	0.9	0.5
	Endometrial serous carcinoma	82	66	100.0	0.0	0.0	0.0
	Carcinosarcoma of the uterus	48	43	90.7	7.0	2.3	0.0
	Endometrial carcinoma, high grade, G3	13	12	83.3	8.3	0.0	8.3
	Endometrial clear cell carcinoma	8	7	71.4	28.6	0.0	0.0
	Endometrioid carcinoma of the ovary	110	84	90.5	8.3	1.2	0.0
	Serous carcinoma of the ovary	559	360	89.4	10.3	0.3	0.0
	Mucinous carcinoma of the ovary	96	68	97.1	2.9	0.0	0.0
	Clear cell carcinoma of the ovary	50	39	92.3	7.7	0.0	0.0
	Carcinosarcoma of the ovary	47	37	89.2	8.1	2.7	0.0
	Granulosa cell tumor of the ovary	37	36	0.0	0.0	2.8	97.2
	Leydig cell tumor of the ovary	4	4	0.0	0.0	0.0	100.0
	Sertoli cell tumor of the ovary	1	1	0.0	0.0	100.0	0.0
	Sertoli Leydig cell tumor of the ovary	3	3	0.0	0.0	0.0	100.0
	Steroid cell tumor of the ovary	3	3	0.0	0.0	0.0	100.0
	Brenner tumor	41	37	100.0	0.0	0.0	0.0
**Tumors of the breast**	Invasive breast carcinoma of no special type	80	74	95.9	4.1	0.0	0.0
	Lobular carcinoma of the breast	122	98	99.0	1.0	0.0	0.0
	Medullary carcinoma of the breast	15	15	100.0	0.0	0.0	0.0
	Tubular carcinoma of the breast	18	15	100.0	0.0	0.0	0.0
	Mucinous carcinoma of the breast	22	15	100.0	0.0	0.0	0.0
	Phyllodes tumor of the breast	50	48	100.0	0.0	0.0	0.0
**Tumors of the digestive system**	Adenomatous polyp, low-grade dysplasia	50	45	100.0	0.0	0.0	0.0
	Adenomatous polyp, high-grade dysplasia	50	47	100.0	0.0	0.0	0.0
	Adenocarcinoma of the colon	2482	1960	97.0	2.7	0.3	0.1
	Gastric adenocarcinoma, diffuse type	176	130	100.0	0.0	0.0	0.0
	Gastric adenocarcinoma, intestinal type	174	154	98.1	1.3	0.0	0.6
	Gastric adenocarcinoma, mixed type	62	43	97.7	2.3	0.0	0.0
	Adenocarcinoma of the esophagus	83	82	100.0	0.0	0.0	0.0
	Squamous cell carcinoma of the esophagus	76	71	97.2	2.8	0.0	0.0
	Squamous cell carcinoma of the anal canal	89	79	98.7	1.3	0.0	0.0
	Cholangiocarcinoma	113	95	78.9	13.7	1.1	6.3
	Hepatocellular carcinoma	50	48	100.0	0.0	0.0	0.0
	Ductal adenocarcinoma of the pancreas	612	322	97.5	2.2	0.3	0.0
	Pancreatic/Ampullary adenocarcinoma	89	57	100.0	0.0	0.0	0.0
	Acinar cell carcinoma of the pancreas	16	15	20.0	33.3	20.0	26.7
	Gastrointestinal stromal tumor (GIST)	50	47	100.0	0.0	0.0	0.0
**Tumors of the urinary system**	Non-invasive papillary urothelial carcinoma, pTa G2 low grade	177	133	100.0	0.0	0.0	0.0
	Non-invasive papillary urothelial carcinoma, pTa G2 high grade	141	106	99.1	0.9	0.0	0.0
	Non-invasive papillary urothelial carcinoma, pTa G3	219	163	99.4	0.6	0.0	0.0
	Urothelial carcinoma, pT2-4 G3	1318	1047	97.0	2.7	0.1	0.2
	Squamous cell carcinoma of the bladder	22	21	100.0	0.0	0.0	0.0
	Small cell neuroendocrine carcinoma of the bladder	23	22	95.5	4.5	0.0	0.0
	Sarcomatoid urothelial carcinoma	25	11	100.0	0.0	0.0	0.0
	Urothelial carcinoma of the kidney pelvis	62	62	98.4	1.6	0.0	0.0
	Clear cell renal cell carcinoma	857	770	97.7	1.2	0.1	1.0
	Papillary renal cell carcinoma	255	223	99.6	0.4	0.0	0.0
	Clear cell (tubulo) papillary renal cell carcinoma	21	20	70.0	5.0	5.0	20.0
	Chromophobe renal cell carcinoma	131	113	98.2	1.8	0.0	0.0
	Oncocytoma	177	156	100.0	0.0	0.0	0.0
**Tumors of the male genital organs**	Adenocarcinoma of the prostate, Gleason 3+3	83	83	100.0	0.0	0.0	0.0
	Adenocarcinoma of the prostate, Gleason 4+4	80	79	100.0	0.0	0.0	0.0
	Adenocarcinoma of the prostate, Gleason 5+5	85	85	100.0	0.0	0.0	0.0
	Adenocarcinoma of the prostate (recurrence)	258	217	99.1	0.9	0.0	0.0
	Small cell neuroendocrine carcinoma of the prostate	19	17	100.0	0.0	0.0	0.0
	Seminoma	621	591	98.0	1.7	0.2	0.2
	Embryonal carcinoma of the testis	50	22	100.0	0.0	0.0	0.0
	Leydig cell tumor of the testis	30	30	0.0	0.0	0.0	100.0
	Sertoli cell tumor of the testis	2	2	0.0	0.0	50.0	50.0
	Sex cord stromal tumor of the testis	1	1	0.0	0.0	0.0	100.0
	Spermatocytic tumor of the testis	1	1	100.0	0.0	0.0	0.0
	Yolk sac tumor	50	25	100.0	0.0	0.0	0.0
	Teratoma	50	34	91.2	8.8	0.0	0.0
	Squamous cell carcinoma of the penis	80	80	98.8	0.0	1.3	0.0
**Tumors of endocrine organs**	Adenoma of the thyroid gland	113	99	99.0	1.0	0.0	0.0
	Papillary thyroid carcinoma	391	250	84.4	13.2	2.4	0.0
	Follicular thyroid carcinoma	154	109	97.2	2.8	0.0	0.0
	Medullary thyroid carcinoma	111	94	94.7	5.3	0.0	0.0
	Parathyroid gland adenoma	43	42	100.0	0.0	0.0	0.0
	Anaplastic thyroid carcinoma	45	43	95.3	4.7	0.0	0.0
	Adrenal cortical adenoma	50	28	7.1	14.3	32.1	46.4
	Adrenal cortical carcinoma	26	25	20.0	36.0	20.0	24.0
	Phaeochromocytoma	50	49	93.9	4.1	2.0	0.0
	Appendix, neuroendocrine tumor (NET)	22	13	84.6	15.4	0.0	0.0
	Colorectal, neuroendocrine tumor (NET)	12	8	75.0	25.0	0.0	0.0
	Ileum, neuroendocrine tumor (NET)	49	42	92.9	7.1	0.0	0.0
	Lung, neuroendocrine tumor (NET)	19	17	100.0	0.0	0.0	0.0
	Pancreas, neuroendocrine tumor (NET)	97	84	79.8	8.3	2.4	9.5
	Colorectal, neuroendocrine carcinoma (NEC)	12	7	100.0	0.0	0.0	0.0
	Gallbladder, neuroendocrine carcinoma (NEC)	4	3	66.7	33.3	0.0	0.0
	Pancreas, neuroendocrine carcinoma (NEC)	14	14	85.7	14.3	0.0	0.0
**Tumors of hematopoietic and lymphoid tissues**	Hodgkin Lymphoma	103	95	100.0	0.0	0.0	0.0
	Small lymphocytic lymphoma, B-cell type (B-SLL/B-CLL)	50	50	100.0	0.0	0.0	0.0
	Diffuse large B cell lymphoma (DLBCL)	113	113	99.1	0.0	0.0	0.9
	Follicular lymphoma	88	87	100.0	0.0	0.0	0.0
	T-cell Non Hodgkin lymphoma	25	25	96.0	0.0	0.0	4.0
	Mantle cell lymphoma	18	18	100.0	0.0	0.0	0.0
	Marginal zone lymphoma	16	15	100.0	0.0	0.0	0.0
	Diffuse large B-cell lymphoma (DLBCL) in the testis	16	16	100.0	0.0	0.0	0.0
	Burkitt lymphoma	5	2	100.0	0.0	0.0	0.0
**Tumors of soft tissue and bone**	Tendosynovial giant cell tumor	45	37	100.0	0.0	0.0	0.0
	Granular cell tumor	53	31	0.0	0.0	12.9	87.1
	Leiomyoma	50	48	100.0	0.0	0.0	0.0
	Leiomyosarcoma	87	79	94.9	3.8	1.3	0.0
	Liposarcoma	132	107	100.0	0.0	0.0	0.0
	Malignant peripheral nerve sheath tumor (MPNST)	13	11	100.0	0.0	0.0	0.0
	Myofibrosarcoma	26	26	100.0	0.0	0.0	0.0
	Angiosarcoma	73	55	72.7	14.5	12.7	0.0
	Angiomyolipoma	91	65	100.0	0.0	0.0	0.0
	Dermatofibrosarcoma protuberans	21	14	100.0	0.0	0.0	0.0
	Ganglioneuroma	14	14	100.0	0.0	0.0	0.0
	Neurofibroma	117	111	100.0	0.0	0.0	0.0
	Sarcoma, not otherwise specified (NOS)	74	66	98.5	0.0	0.0	1.5
	Paraganglioma	41	41	92.7	2.4	2.4	2.4
	Ewing sarcoma	23	13	100.0	0.0	0.0	0.0
	Rhabdomyosarcoma	6	5	100.0	0.0	0.0	0.0
	Schwannoma	121	115	97.4	2.6	0.0	0.0
	Synovial sarcoma	12	8	75.0	12.5	12.5	0.0
	Osteosarcoma	43	28	100.0	0.0	0.0	0.0
	Chondrosarcoma	38	15	100.0	0.0	0.0	0.0
	Rhabdoid tumor	5	5	100.0	0.0	0.0	0.0

**Figure 2:**
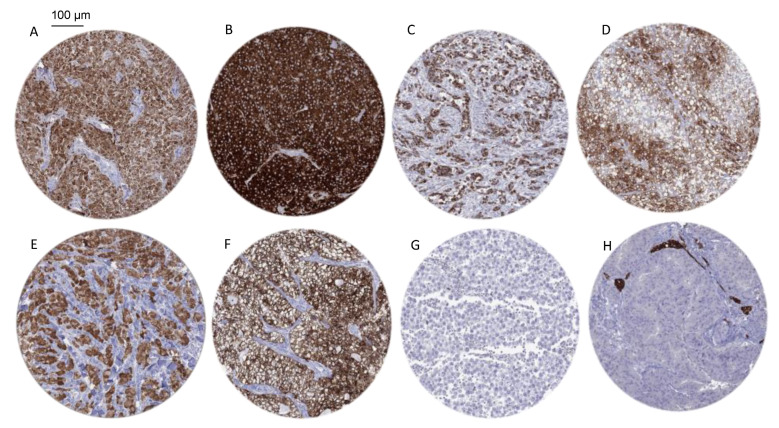
INHA immunostaining in cancer. The panels show a cytoplasmatic INHA immunostaining of variable intensity in samples from a granulosa cell tumor of the ovary (**A**), a Leydig cell tumor (**B**) and Sertoli cell tumor of the testis (**C**), an adrenocortical carcinoma (**D**), a granular cell tumor from the floor of mouth (**E**), and a clear cell carcinoma of the kidney (**F**). INHA immunostaining is absent, however, in a testicular seminoma (**G**) and in tumor cells of a high-grade serous carcinoma of the ovary which contains INHA-positive stroma cells (**H**).

Representative images of INHA positive tumors are shown in Figure 2.

## 4. Discussion

More than 12,000 tumors were successfully analyzed in this study. Considering the large scale of our study, our assay was extensively validated by comparing our IHC findings in normal tissues with RNA data derived from three different publicly accessible databases [33,34,35,36] and immunostaining data obtained by a second independent anti-INHA antibody. This approach has been suggested by the international working group for antibody validation (IWGAV) for the validation of IHC assays designed for formalin-fixed tissues [33]. To ensure as broad as possible a range of proteins to be tested for possible cross-reactivity, 76 different normal tissue categories were included in this analysis. The fact that INHA immunostaining was only seen in the testis, ovary, placenta, adrenal glands, pancreas, and the adenohypophysis supports the validity of our assay because INHA RNA expression was also detected in these organs. Additional validation comes from the staining of identical cell types such as the Sertoli and Leydig cells of the testis, corpus luteum, follicular, granulosa, and stroma cells of the ovary, cyto- and syncytiotrophoblast, as well as chorion cells, amnion, and decidua cells of the placenta, and adrenocortical cells, as well as scattered epithelial cells in the pancreas by an independent second antibody (Figure S1).

INHA protein expression is generally considered an important diagnostic feature for adrenocortical tumors and granular cell tumors, as well as sex cord stromal tumors of the testis and the ovary [28,37,38,39]. The fact that the vast majority of these tumors showed a strong INHA expression in our study is thus consistent with the literature and confirms the utility of INHA immunostaining for supporting these diagnoses [40]. The extended analysis of 134 different tumor entities for INHA expression, including more than 80 tumor types and subtypes that had not been examined thus far for INHA expression showed, however, that INHA expression can occur in a much broader spectrum of tumors.

A small fraction of tumors of various categories showed a strong INHA positivity that was comparable to the expression levels of adrenocortical, granular cell, and sex cord stromal tumors. These especially included multiple cases of acinar cell carcinoma of the pancreas. This tumor entity had not been analyzed thus far for INHA expression. It constitutes a rare but highly malignant tumor derived from pancreatic acinar cells, a cell type showing low level INHA protein expression in our normal tissue screening. Other tumor entities which can show high-level INHA expression include, for example, neuroendocrine tumors of the pancreas, cholangiocarcinoma, adenocarcinoma of the lung, gastric adenocarcinoma, and clear cell renal cell carcinoma. The ability of renal carcinomas to highly express INHA is of particular relevance because INHA immunohistochemistry is used as a tool to distinguish normal or neoplastic adrenal tissue from clear cell renal cell carcinomas, as these entities may be difficult to distinguish by morphology alone [41,42,43]. Our data suggest that this clinically important distinction should not be solely based on the identification of high-level INHA expression in a tissue in question.

The majority of our INHA-positive cases showed INHA immunostaining in only a small fraction of tumor cells, often in the range of 1–10% of tumor cells, while adrenocortical, granular cell, and sex cord stromal tumors usually showed a moderate to strong INHA positivity in all or almost all tumor cells. These findings demonstrate that a focal weak to moderate INHA immunostaining should not be diagnostically overinterpreted. The biological role of focal low-level or even diffuse high-level INHA expression in cancers derived from cells that normally do not express INHA is unknown. The observation would, however, be consistent with a paracrine role of INHA in these tumors. INHA has recently been suggested as a novel paracrine factor for tumor angiogenesis and metastasis based on in vitro experiments demonstrating that tumor-cell-derived INHA can induce the growth of cultured endothelial cells through a signaling pathway involving the TGF beta co-receptor endoglin and its downstream activators of angiogenesis, ALK1 and SMAD1/5 [44]. The authors also show RNA data indicating a poor clinical outcome of INHA-positive tumors in ovarian cancers and renal cell carcinomas [44].

Due to the rareness of immunohistochemically detectable INHA expression in most cancer types, we were only able to compare INHA immunostaining data with available clinical data in neuroendocrine tumors, clear cell renal cell carcinoma, and colorectal adenocarcinoma. The fact that positive INHA immunostaining was marginally related to features of cancer aggressiveness in all these cancer types would be consistent with the notion that the neo-expression of INHA in cancers could exert a tumor-promoting effect, potentially through a paracrine activity of secreted INHA. A strong expression of INHA in tumor adjacent stroma cells observed in a fraction of otherwise INHA-negative ovarian carcinomas would also be consistent with the paracrine stimulation of tumor cell growth.

**Figure 3:**
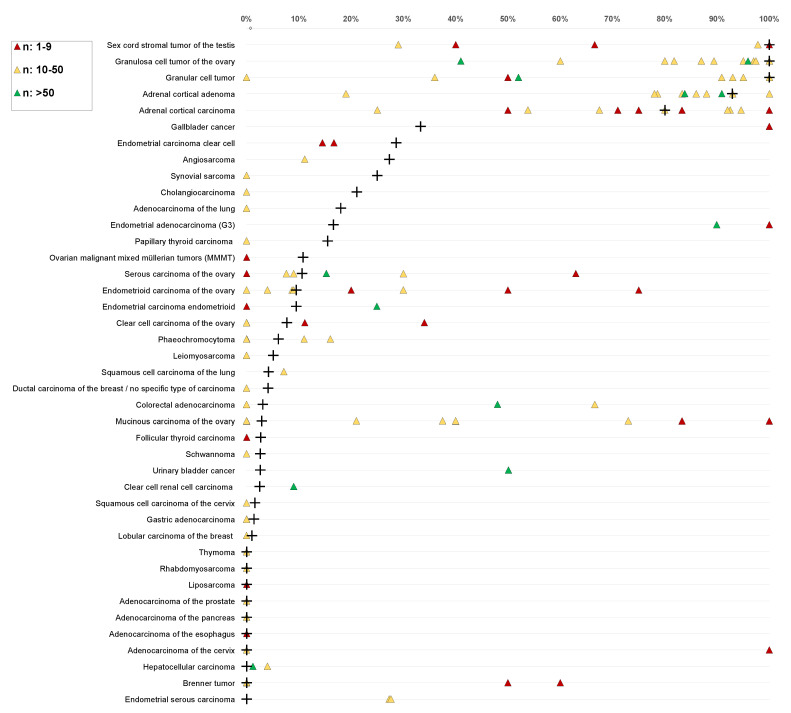
INHA data from previous literature. The colors of the triangles represent the numbers of analyzed tumors in these studies: red: n = 1–9, yellow: n = 10–50, green: n >50. + indicates results of this study. For raw data and references, see Table S1.

Our data provide a comprehensive ranking list of tumors according to their INHA expression across a large variety of tumor entities. It is almost certain that the use of different protocols, antibodies, interpretation criteria, and thresholds to define “positivity” have jointly caused the high diversity of literature data on INHA expression in tumors (summarized in Figure 3).

The positivity rates described in the present study are thus specific to the reagents and protocols used in our laboratory. In contrast to previous studies using other reagents, relevant INHA1 immunostaining was not observed in adenocarcinomas of the esophagus or urothelial carcinomas, nor in mucinous, serous, or endometroid ovarian carcinomas. It is expected that different experimental conditions could change the INHA positivity rates—especially in tumors with low expression levels—but this would have little impact on the tumor ranking based on the INHA positivity rates.

## 5. Conclusions

Our data corroborate that INHA is commonly expressed in various types of sex cord stromal tumors and granular cell tumors, as well as adrenal cortical neoplasms. Considering the fact that INHA expression can also be found in 60 other tumor entities, including 15 entities with a fraction of strongly positive cancers, INHA immunohistochemistry should only be applied as a part of a panel for the distinction of tumor entities. While the data from ourselves and others suggest a potential link between INHA expression and increased aggressiveness in various cancer types, the functional role of INHA in these tumors awaits further investigation.

## Figures and Tables

**Table 2:** INHA immunostaining and cancer phenotype.

				Inhibin Alpha (INHA) IHC Result				
			n	Negative (%)	Weak (%)	Moderate (%)	Strong (%)	*p*
Colon adenocarcinoma	Primary tumor	pT1	69	100.0	0.0	0.0	0.0	0.2243
		pT2	372	98.7	1.1	0.3	0.0	
		pT3	1042	96.4	3.3	0.3	0.1	
		pT4	363	96.4	3.3	0.3	0.0	
	Regional lymph nodes	pN0	982	97.9	1.8	0.3	0.0	0.0494
		pN+	860	95.9	3.7	0.2	0.1	
	Tumor localization	left colon	930	97.2	2.8	0.0	0.0	0.2291
		right colon	385	97.7	2.1	0.3	0.0	
	MMR status	defective	71	97.2	2.8	0.0	0.0	0.8868
		proficient	907	97.5	2.5	0.0	0.0	
	RAS mutation status	mutated	226	94.7	5.3	0.0	0.0	0.0814
		wildtype	292	97.6	2.4	0.0	0.0	
	BRAF mutation status	mutated	10	100.0	0.0	0.0	0.0	0.5117
		wildtype	89	97.8	2.2	0.0	0.0	
Clear cell renal cell carcinomas	ISUP grade	1	235	99.6	0.0	0.0	0.4	0.0801
		2	231	96.1	2.2	0.0	1.7	
		3	207	97.6	1.4	0.5	0.5	
		4	43	95.3	4.7	0.0	0.0	
	Fuhrmann grade	1	42	100.0	0.0	0.0	0.0	0.2987
		2	422	97.6	1.2	0.0	1.2	
		3	209	98.1	1.0	0.5	0.5	
		4	52	94.2	5.8	0.0	0.0	
	Thoenes’ grade	1	267	99.3	0.7	0.0	0.0	0.0498
		2	390	96.9	1.3	0.3	1.5	
		3	68	95.6	4.4	0.0	0.0	
	UICC stage	1	339	98.2	0.9	0.0	0.9	0.2994
		2	37	100.0	0.0	0.0	0.0	
		3	91	97.8	1.1	1.1	0.0	
		4	74	95.9	4.1	0.0	0.0	
	Primary tumor	1	438	97.9	1.1	0.0	0.9	0.3615
		2	73	98.6	0.0	0.0	1.4	
		3–4	210	96.7	2.4	0.5	0.5	
	Regional lymph nodes	0	122	96.7	0.8	0.8	1.6	0.4863
		≥1	18	94.4	5.6	0.0	0.0	
	Distant metastasis	0	108	98.1	0.9	0.0	0.9	0.225
		≥1	75	96.0	4.0	0.0	0.0	
Neuroendocrine tumors	Primary tumor	pT1	24	91.7	8.3	0.0	0.0	0.3256
		pT2	25	88.0	8.0	0.0	4.0	
		pT3	37	78.4	8.1	5.4	8.1	
		pT4	28	92.9	7.1	0.0	0.0	
	Regional lymph nodes	pN0	41	92.7	7.3	0.0	0.0	0.0824
		pN+	58	82.8	6.9	3.4	6.9	

## Data Availability

All data generated or analyzed during this study are included in this published article.

## Supplementary Materials

The following supporting information can be downloaded at: https://www.mdpi.com/article/10.3390/biomedicines10102507/s1, Figure S1: IHC validation by comparison of antibodies. The panels show a concordance of immunostaining results obtained by two independent INHA antibodies. Using MSVA-561R, significant cytoplasmic staining is seen in adrenocortical cells (**A**), Sertoli and Leydig cells of the testis (**B**), the corpus luteum (**C**) and theca cells (**D**) of the ovary, decidua cells in the pregnant uterus (**E**), and in trophoblast cells of the first trimester placenta (**F**). Using anti-inhibin α (clone R1), comparable staining is seen in the adrenal gland (**G**), testis (**H**), corpus luteum (**I**) and theca cells (**K**) of the ovary, decidua cells (**L**), and in the placenta (**M**). The images A–F and G–M are from consecutive tissue sections. Table S1: List of the references and raw data used to create Figure 3.
