# Supplementary material for: Inhibin Alpha Expression in Human Tumors: A Tissue Microarray Study on 12,212 Tumors
Source: Biomedicines. 2022 Oct 7;10(10):2507. doi: 10.3390/biomedicines10102507 (PMC9598868; doi:10.3390/biomedicines10102507)
Supplement: Supplementary file 1 [file biomedicines-10-02507-s001.zip › Supplementary Figure S1.pdf]

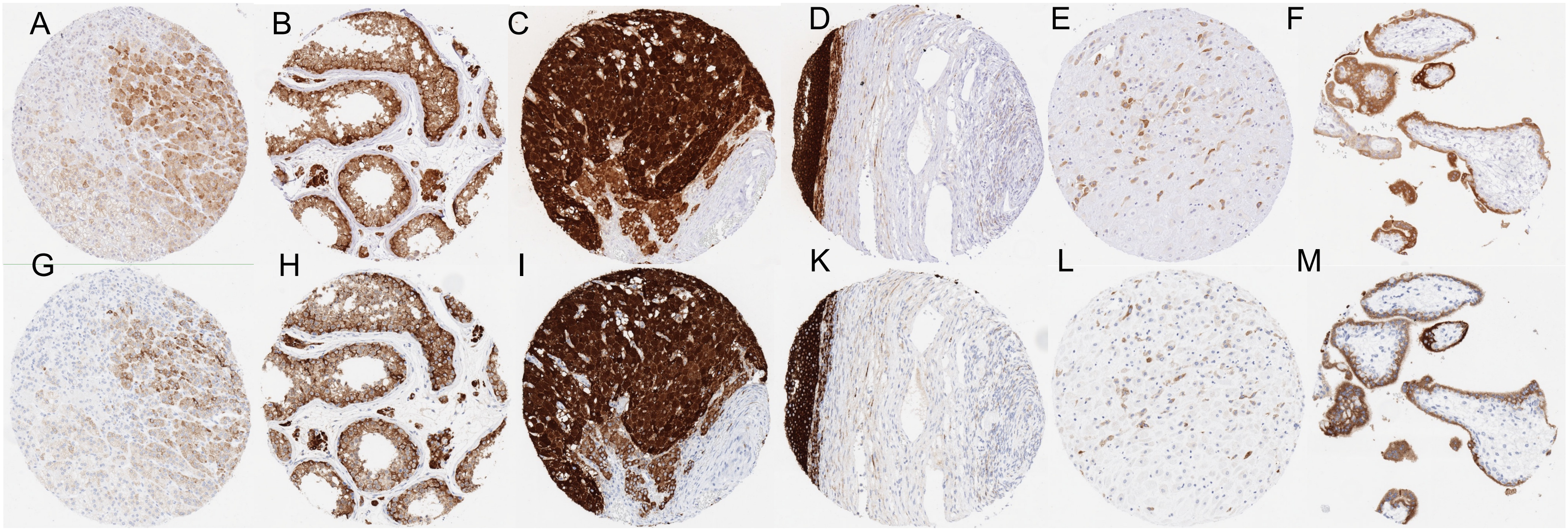

Figure S1: IHC validation by comparison of antibodies. The panels show a concordance of immunostaining results obtained by two independent INHA antibodies. Using MSVA-561R, significant cytoplasmic staining is seen in adrenocortical cells (**A**), Sertoli and Leydig cells of the testis (**B**), the corpus luteum (**C**) and theca cells (**D**) of the ovary, decidua cells in the pregnant uterus (**E**), and in trophoblast cells of the first trimester placenta (**F**). Using anti-inhibin α (clone R1), comparable staining is seen in the adrenal gland (**G**), testis (**H**), corpus luteum (**I**) and theca cells (**K**) of the ovary, decidua cells (**L**), and in the placenta (**M**). The images A–F and G–M are from consecutive tissue sections.
